# Long-term outcomes of dupilumab therapy in severe asthma: A retrospective, multicenter, real-world study

**DOI:** 10.1016/j.jacig.2025.100533

**Published:** 2025-07-08

**Authors:** Carlo Mümmler, Alexandra Lenoir, Jeremias Götschke, Michael Gerckens, Merle Kaiser, Moritz Kayser, Nora Drick, Hendrik Suhling, Leonie Biener, Carmen Pizarro, Dirk Skowasch, Nikolaus Kneidinger, Jürgen Behr, Katrin Milger

**Affiliations:** a Department of Medicine V, LMU University Hospital, Comprehensive Pneumology Center (CPC), LMU Munich, Member of the German Center of Lung Research (DZL), Munich, Germany; b Institute of Lung Health and Immunity (LHI), Comprehensive Pneumology Center (CPC), Helmholtz Munich, Member of the German Center of Lung Research (DZL), Munich, Germany; c Department of Respiratory Medicine and Infectious Diseases, Hannover Medical School, and Biomedical Research in Endstage and Obstructive Lung Disease Hannover (BREATH), German Center for Lung Research (DZL), Hannover, Germany; d Department of Microbiology and Immunology, Weill Cornell Medicine, New York, New York; e Department of Internal Medicine II, Cardiology, Pneumology, Angiology, University Hospital Bonn, Bonn, Germany; f Department of Internal Medicine, Division of Respiratory Medicine, Lung Research Cluster, Medical University of Graz, Graz, Austria

**Keywords:** Severe asthma, dupilumab, long-term, antibody, real-world

## Abstract

**Background:**

Dupilumab is an IL-4Rα antibody approved for treatment of severe asthma. Real-world data on the continuation and cessation patterns of dupilumab and long-term treatment efficacy are scarce.

**Objective:**

We sought to analyze real-world, long-term treatment outcomes and to evaluate trajectories of patients continuing or discontinuing dupilumab therapy over a 3-year period.

**Methods:**

This multicenter, retrospective, real-world cohort study included patients with severe asthma who started dupilumab before March 2021. Data on asthma control, medication, lung function, and annualized exacerbation rates were collected at baseline and 3, 12, and 36 months after initiation of dupilumab therapy. Asthma remission was assessed at 12 months and 36 months after dupilumab initiation.

**Results:**

Of 160 included patients, 95 patients (59%) continued dupilumab therapy for 36 months; 65 patients (41%) discontinued therapy after a median time of therapy of 8 months. Patients who continued dupilumab for 36 months had significant reductions in annual exacerbations (−1; *P* < .0001) and oral corticosteroid dose (−5.5 mg/day; *P* < .001) as well as significant improvements in asthma control (asthma control test +5; *P* < .0001) and lung function (percent predicted of FEV_1_ +7%; *P* < .001) compared with baseline. Of patients who continued dupilumab, 30% achieved remission at 12 months, and 26% achieved remission at 36 months. Of the 65 patients who discontinued therapy, 55 switched to another antibody, and 10 did not receive further antibody treatment.

**Conclusions:**

Dupilumab represents an effective long-term treatment option for patients with severe asthma, with sustained treatment effects up to 36 months. Importantly, a relevant proportion of patients achieved remission in this pretreated population.

Asthma is a highly prevalent chronic respiratory condition and represents a substantial burden on individuals and health care systems.^1^ Severe asthma is defined as asthma that remains uncontrolled despite optimized treatment with high-dose inhaled corticosteroid (ICS)/long-acting β-agonist (LABA) or that requires high-dose ICS/LABA to prevent it from becoming uncontrolled.^2^^,^^3^ Patients with severe asthma experience recurrent exacerbations, debilitating symptoms, impaired lung function, and long-term side effects of corticosteroid therapy.^2^^,^^3^

Central to asthma pathophysiology is airway inflammation, driven by type 2 innate lymphoid cells that, in response to epithelial cell damage or environmental triggers, release IL-25, IL-33, and thymic stromal lymphopoietin.^4^ These cytokines promote differentiation of naive T cells into T_H_2 cells, mainly driven by IL-4. Subsequently, type 2 cells migrate to the airway mucosa and secrete key cytokines that perpetuate the inflammatory cascade: IL-5 recruits eosinophilic granulocytes from the bone marrow, whereas IL-4 not only sustains the differentiation of T cells, but also drives IgE class switching in B cells.^4^ IL-13 contributes to mucus hypersecretion, airway smooth muscle contraction, and airway remodeling.^4^ The discovery of these pathways led to the 6 currently approved mAbs that offer personalized treatment strategies.^1^^,^^5^

Dupilumab, a human mAb that binds to IL-4Rα, inhibits both IL-4 and IL-13 signaling and reduces asthma exacerbations, decreases oral corticosteroid (OCS) dosage, and improves lung function and symptom control in patients with asthma.^6^^,^^7^ Dupilumab has been approved by the US Food and Drug Administration and the European Medicines Agency for patients with severe asthma characterized by elevated blood eosinophil counts (BECs) (≥150 /μL) and/or fraction of exhaled nitric oxide (Feno) (≥25 ppb).

Although randomized controlled trials (RCTs) and extension studies such as TRAVERSE have demonstrated dupilumab efficacy over up to 3 years, real-world long-term data remain limited to 2 years.^8^^,^^9^ Importantly, current guidelines recommend a switching of biological therapy when therapeutic goals are not achieved,^3^^,^^10^ and thus an increasing number of patients with severe asthma have received more than 1 biological therapy.^11^^,^^12^ Studies such as TRAVERSE that included only biologic-naive patients thus do not fully capture the current real-world situation.^8^ Remission has become the key treatment goal with the most important components being absence of OCS use, exacerbations, and asthma symptoms together with stable or improved lung function.^10^^,^^13^ It remains uncertain to what extent the therapeutic effects of dupilumab are sustained over longer treatment periods and how many patients achieve remission on dupilumab in a pretreated real-world patient collective.

To close this gap, we performed a multicenter retrospective cohort study of patients with severe asthma in Germany in whom dupilumab treatment was initiated before March 2021. We assessed treatment continuation and cessation patterns and evaluated long-term treatment outcomes including remission criteria in a real-world patient collective.

## Methods

### Ethics

All patients gave written informed consent for inclusion in the German Asthma Net (GAN) Severe Asthma Registry, which was approved by the Ethics Committee of the University of Mainz and local institutional review boards at each institution and is conducted in accordance with the principles of the Declaration of Helsinki. The GAN prospectively collects routine clinical parameters of patients with severe asthma at baseline and follow-up examinations.

### Patient cohort

Three outpatient centers from the GAN specializing in management of severe adult asthma participated in this retrospective study (Medizinische Hochschule Hannover, Universitätsklinikum Bonn, and Ludwig-Maximilians-Universität München).

### Inclusion and exclusion criteria

Inclusion criteria were severe asthma as defined by European Respiratory Society/American Thoracic Society guidelines and the initiation of dupilumab therapy in March 2021 to ensure 3-year follow-up.^2^^,^^14^ Patients younger than 18 years old and patients with dual biological therapies were excluded. Medication was prescribed by the treating physician in routine clinical practice with a loading dose of 600 or 400 mg of dupilumab via subcutaneous injection and subsequent 300- or 200-mg subcutaneous injections every 2 weeks.

### Patient data

The data were collected as part of routine clinical practice until December 2024 and were assessed retrospectively at baseline before dupilumab therapy and at 3 months, 12 months, and 36 months after dupilumab initiation. Patient characteristics, asthma exacerbations, medication, pulmonary function tests, and laboratory tests were collected from electronic health records. Early-onset asthma was defined as age of onset 18 years or younger, and adult-onset asthma was defined as age of onset older than 18 years.^15^ Annualized exacerbation rates were calculated by dividing the total number of exacerbations during the observation period.

### Asthma remission

Asthma remission was defined according to the S2k-Leitlinie zur fachärztlichen Diagnostik und Therapie von Asthma (S2k Guideline for Respiratory Specialists for the Diagnosis and Treatment of Asthma) guideline by fulfilling all 4 of the following criteria: asthma control test (ACT) score ≥ 20 points, no exacerbations in previous 12 months, no OCS therapy, and stable lung function (FEV_1_).^10^ An exacerbation was defined as an acute worsening of the patient’s symptoms and/or lung function necessitating OCS burst therapy with at least 20 mg/day for at least 3 consecutive days.^3^ Stable lung function was defined as an FEV_1_ value that was equal to or greater compared with the baseline measurement.

### Statistical analysis

A Sankey diagram was generated using SankeyMATIC (https://sankeymatic.com/). Categorical data are presented as absolute numbers and percentages. Continuous variables are presented as mean ± SD if normally distributed or median (interquartile range [IQR]) if non-normally distributed. Differences between groups were analyzed using χ^2^ test or Fisher exact test for categorical data. Paired or unpaired *t* tests were used for normally distributed data. For non-normally distributed data, Wilcoxon signed-rank test was used for paired data, and Mann-Whitney *U* test was used for unpaired data. For multiple comparisons and within-subject correlations in longitudinal data with partially missing values, we used a mixed-effects model available in GraphPad Prism (GraphPad Software, Inc, Boston, Mass). This model assumes a compound symmetry covariance structure and is fit using restricted maximum likelihood. Binary logistic regression analysis was performed on the end point of achieving remission. A *P* value < .05 was considered statistically significant. All statistical analyses were performed using IBM SPSS Statistics 28 (IBM Corp, Armonk, NY) and GraphPad Prism 10.1.1.

## Data availability

Data are available from the corresponding author on reasonable request.

## Results

### Patient cohort

**Fig 1:**
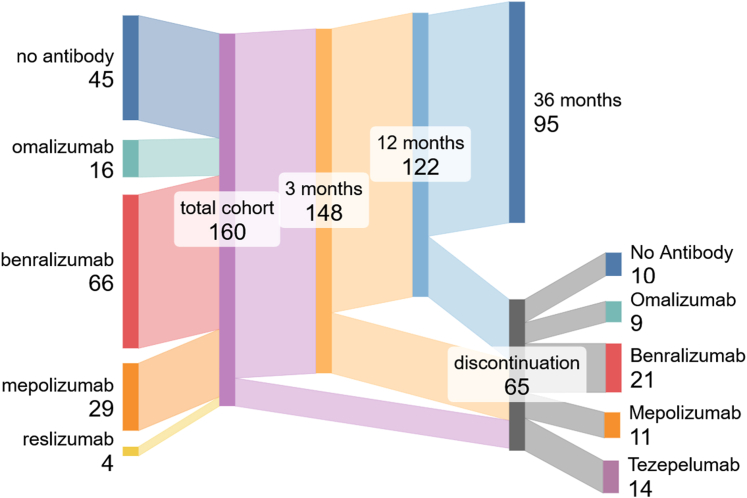
Sankey-Plot illustrating patient trajectories before initiation and during dupilumab therapy.

We identified 160 patients with severe asthma from outpatient clinics at Universitätsklinikum Bonn (n = 34), Medizinische Hochschule Hannover (n = 56), and Ludwig-Maximilians-Universität Munich (n = 70) who had started dupilumab therapy before March 2021 and had follow-up data available. A total of 95 patients (59%) continued therapy for more than 36 months, whereas 65 patients (41%) discontinued dupilumab therapy during the 36-month interval. Of the 65 patients who discontinued therapy, 21 patients were subsequently switched to benralizumab, 14 were switched to tezepelumab, 11 were switched to mepolizumab, 9 were switched to omalizumab, and 10 did not receive further antibody treatment (Fig 1).

### Baseline characteristics of total cohort

Of all patients, 54% were female and median BMI of the cohort was 28 kg/m^2^. A relevant number of patients experienced comorbidities, with the most frequent being chronic rhinosinusitis with nasal polyps (CRSwNP) (45%), allergic rhinitis (34%), and chronic rhinosinusitis without nasal polyps (28%). A relevant number of patients were former smokers (41%) with a median of 10 pack-years (Table I). One-third of patients had an early-onset asthma phenotype, and two-thirds had an adult-onset asthma phenotype. Prior to dupilumab initiation, asthma was uncontrolled with a median ACT score of 14, a median of 1 annual exacerbation, and continuous OCS therapy in 28% of patients with a median OCS dosage of 8.8 mg. Blood eosinophils were elevated with a median of 270/μL when considering only patients without previous antibody therapy during 2 months before dupilumab initiation. Further, Feno was elevated with a median of 43 ppb and IgE of 145 IU/mL. Notably, the majority of patients had received a previous antibody therapy (41% received benralizumab; 18%, mepolizumab; 10%, omalizumab; and 3%, reslizumab) (Table II).

**Table I:** Clinical characteristics of total cohort

Variable	Value
No. of patients	160
Age (y), median (IQR)	57 (48-62)
Female, no. (%)	86 (54)
BMI (kg/m^2^), median (IQR)	28 (23-33)
Age at diagnosis (y), median (IQR)	30 (10-42)
Time since asthma diagnosis (y), median (IQR)	23 (11-37)
Early-onset asthma (age ≤18 y), no. (%)	54 (34)
Adult-onset asthma (age >18 y), no. (%)	106 (66)
Patients with allergies, no. (%)	112 (70)
Former smokers, no. (%)	65 (41)
Pack-years in former smokers, median (IQR)	10 (5-22)
Comorbidities, no. (%)	
CRSwNP	72 (45)
Allergic rhinitis	55 (34)
CRSsNP	44 (28)
Aspirin intolerance	37 (23)
Atopic dermatitis	35 (22)
Steroid-induced side effects	34 (21)
COPD	23 (14)

*BMI,* Body mass index; *COPD,* chronic obstructive pulmonary disease; *CRSsNP,* chronic rhinosinusitis without nasal polyps.

Non-normally distributed data are presented as median (IQR); nominal or ordinal data are presented as no. (%).

**Table II:** Baseline characteristics at dupilumab initiation

Variable	Value
Asthma control, median (IQR)	
ACT	14 (10-18)
Annual exacerbations	1.0 (0-3)
Biomarkers, median (IQR)	
Blood eosinophils (cells/μL)	130 (10-420)
Blood eosinophils (cells/μL) only in patients without antibody therapy 2 mo before dupilumab initiation∗	270 (105-420)
Feno (ppb)	43 (23-79)
IgE (IU/mL)	145 (61-413)
Lung function, mean ± SD	
FEV_1_/FVC%	66 ± 12
FEV_1_ (L)	2.1 ± 0.8
FEV_1_%	69 ± 22
FVC (L)	3.2 ± 1.0
FVC%	82 ± 19
RV (L)	2.9 ± 0.9
RV%	156 ± 45
TLC (L)	6.3 ± 1.3
TLC%	102 ± 15
Previous therapy	
Continuous OCS therapy, no. (%)	44 (28)
OCS dose (mg), median (IQR)†	8.8 (5-10)
LABA/ICS high, no. (%)	150 (94)
LABA/ICS medium, no. (%)	10 (6)
LAMA, no. (%)	137 (86)
LTRA (montelukast), no. (%)	70 (44)
Previous biological therapy, no. (%)	
No antibody therapy	45 (28)
Benralizumab	66 (41)
Mepolizumab	29 (18)
Omalizumab	16 (10)
Reslizumab	4 (3)

*FEV*_*1*_*%,* Percent predicted of FEV_1_; *FVC,* forced vital capacity; *FVC%,* percent predicted of FVC; *LAMA,* long-acting muscarinic antagonist; *LTRA,* leukotriene receptor antagonist; *RV,* residual volume; *RV%,* percent predicted of RV; *TLC,* total lung capacity; *TLC%,* percent predicted of TLC.

Metric data are presented as mean ± SD; non-normally distributed data are presented as median (IQR); nominal or ordinal data are presented as no. (%).

∗ n = 64 patients.

† Only in patients on continuous OCS therapy.

### Patients who discontinued dupilumab

During the 36-month study period, 65 patients (41%) discontinued dupilumab treatment, 12 patients (18%) between initiation and 3-month follow-up, 27 patients (42%) between 3- and 12-month follow-up, and 26 patients (40%) between 12- and 36-month follow-up. The median time point of dupilumab discontinuation was 8 months. Main reasons for discontinuation were insufficient subjective treatment response in 34 patients (52%), insufficient change in lung function in 26 patients (40%), and suspected side effects in 24 patients (37%). Severe hypereosinophilia without organ manifestation led to discontinuation in 11 patients (17%), whereas dupilumab was discontinued owing to hypereosinophilia with organ manifestation in 4 patients (eosinophilic granulomatosis with polyangiitis [EGPA] in 2 patients and eosinophilic pneumonia in 2 patients) (Table III). Patients who stopped dupilumab therapy did not differ from patients who continued dupilumab therapy in regard to baseline characteristics, comorbidities, biomarkers, or previous therapy; however, there was a trend for a lower baseline ACT score among the patients who stopped dupilumab (Table E1 in this article’s Online Repository available at www.jaci-global.org).

**Table III:** Discontinuation patterns

Time point of discontinuation	Value
Duration of dupilumab therapy (mo), median (IQR)	8 (4-22)
Discontinuation between 0 and 3 mo after dupilumab, no. (%)	12 (18)
Discontinuation between 3 and 12 mo after dupilumab, no. (%)	27 (42)
Discontinuation between 12 and 36 months after dupilumab, no. (%)	26 (40)
**Reasons for discontinuation**	
Insufficient subjective treatment response, no. (%)	34 (52)
Insufficient change in lung function, no. (%)	26 (40)
Suspected side effects, no. (%)	24 (37)
Respiratory, no.	6
Skin reactions, no.	6
Hypereosinophilia with organ manifestation, no.	4
Arthralgia, no.	2
Conjunctivitis, no.	2
Other, no.	4
Insufficient reduction in exacerbations, no. (%)	17 (26)
Insufficient reduction in OCS, no. (%)	12 (18)
Severe hypereosinophilia (> 1500/μL), no. (%)	11 (17)
Insufficient response of comorbidities, no. (%)	9 (14)
Persistent allergic symptoms, no. (%)	5 (8)
Insufficient change in symptoms of nasal polyps, no. (%)	4 (6)

Overview of time points and reasons for dupilumab discontinuation in 65 patients who discontinued dupilumab treatment over a 36-month time frame. Multiple reasons per patient were possible. Percentages respective to the total patients who discontinued treatment (n = 65).

### Analysis of patients who continued dupilumab for 36 months

**Fig 2:**
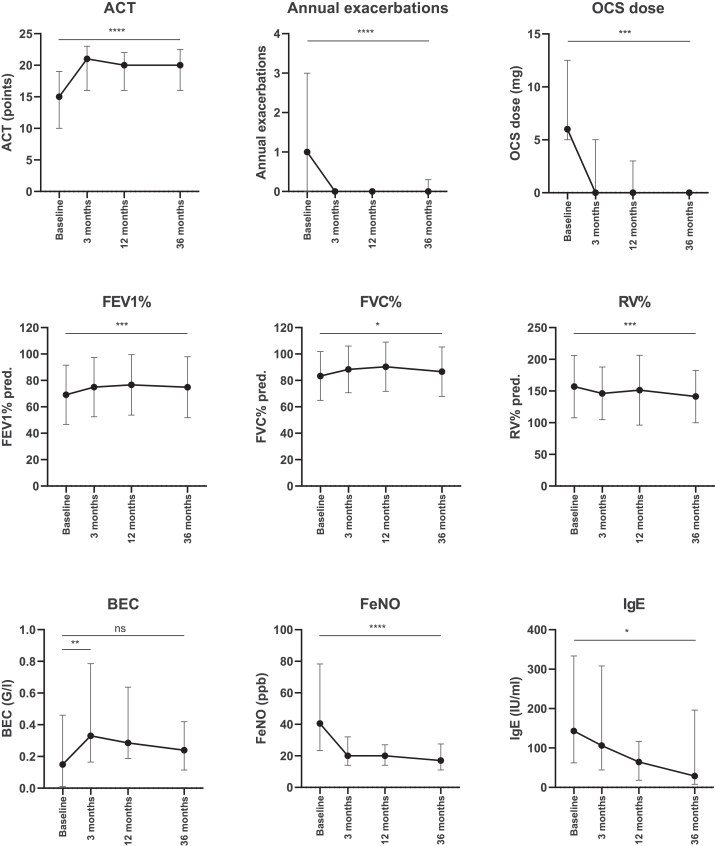
Clinical outcome parameters of 95 patients who continued dupilumab therapy 36 months after initiation. Available data: ACT: n = 95, 84, 87, 95 patients; annual exacerbations: n = 95, 86, 89, 95 patients; OCS dose: n = 95, 90, 92, 95 patients; FEV_1_%: n = 95, 85, 88, 95 patients; FVC%: 95, 84, 88, 95 patients; RV%: n = 94, 83, 88, 92; BEC: n = 91, 65, 54, 53; Feno: 88, 71, 73, 69 patients; IgE: 84, 43, 28, 22 patients. Median and IQR were used for non-normally distributed values (ACT, annual exacerbations, OCS dose, BEC, Feno, IgE), and mean ± SD were used for normally distributed values (FEV_1_%, FVC%, RV%). A mixed-effects model was used for statistical analysis. ∗*P* < .05, ∗∗*P* < .01, ∗∗∗*P* < .001, ∗∗∗∗*P* < .0001. *ns,* Not significant; *RV%,* percent predicted of residual volume.

Patients who continued therapy for 36 months (95 patients, 59%) showed a significant median (IQR) improvement in asthma control as depicted by an increase in ACT score (baseline vs 36 months: 15 points [10-19 points] vs 20 points [16-23 points]; *P* < .0001), decrease in annual exacerbations (baseline vs 36 months: 1 [0-3] vs 0 [0-0]; *P* < .0001), and decrease in OCS dose (baseline vs 36 months: 6 mg [5-13 mg] vs 0 mg [0-0 mg]; *P* < .001). In terms of lung function, there was a significant mean ± SD improvement in percent predicted of FEV_1_ (FEV_1_%) (baseline vs 36 months: 69% ± 22% vs 75% ± 23%; *P* < .001) and percent predicted of forced vital capacity (FVC%) (baseline vs 36 months: 83% ± 19% vs 87% ± 19%; *P* = .04) as well as a significant mean ± SD decrease in percent predicted of residual volume (baseline vs 36 months: 157% ± 49% vs 141% ± 41%; *P* < .001). At the 36-month visit, 35 patients (36%) achieved FEV_1_ improvement of more than 200 mL. Median (IQR) BEC increased at the 3-month follow-up after dupilumab initiation (baseline vs 3 months: 150/μL [10-460/μL] vs 330/μL [165-785/μL]; *P* < .01), but was not significantly different from baseline at the 36-month follow-up (baseline vs 36 months: 150/μL [10-460/μL] vs 240/μL [115-420/μL]; P = .52). Median (IQR) Feno significantly decreased (baseline vs 36 months: 41 ppb [23-78 ppb] vs 17 ppb [11-28 ppb]; *P* < .0001), as did total serum IgE (baseline vs 36 months: 143 IU/mL [62-334 IU/mL] vs 29 IU/mL [8-196 IU/mL], *P* < .05) (Fig 2). Further subgroup analysis was performed for patients who continued therapy for 36 months to evaluate efficacy in ex-smokers (Fig E1, *A*, in the Online Repository at www.jaci-global.org), who had CRSwNP (Fig E1, *B*), and who had early-onset (Fig E1, *C*) or adult-onset asthma (Fig E1, *D*).

### Asthma remission in patients who continued dupilumab for 36 months

**Fig 3:**
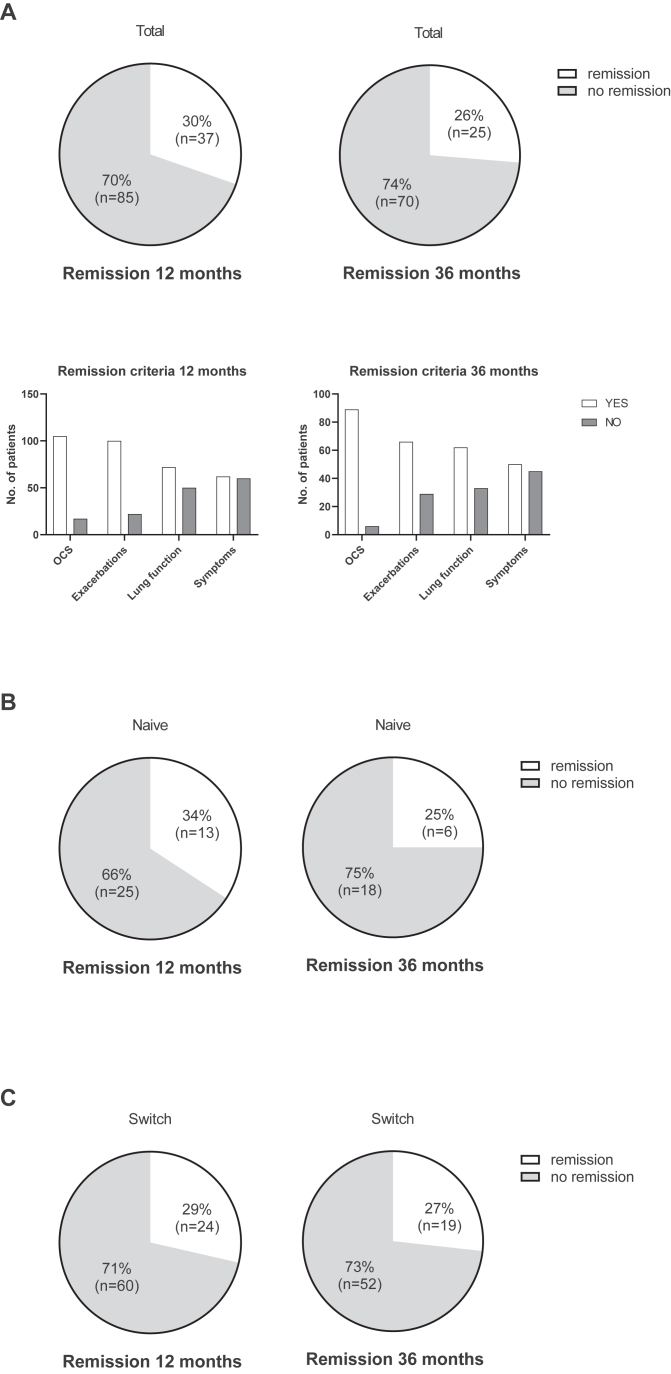
Clinical remission in **(A)** the total cohort at 12 months (122 patients) and 36 months (95 patients) of dupilumab therapy, subfigures indicating the number of patients fulfilling single remission criteria at 12 months and 36 months; **(B)** biologic-naive patients at 12 months (38 patients) and at 36 months (24 patients) of dupilumab therapy; and **(C)** biologic-experienced patients at 12 months (84 patients) and at 36 months (71 patients) of dupilumab therapy.

Asthma remission was analyzed with a 4-strata remission definition (no exacerbations, no OCS, ACT ≥20, lung function stable or better). Of the total cohort of 122 patients at 12 months and 95 patients at 36 months, 37 patients (30%) at 12 months and 25 patients (26%) at 36 months fulfilled remission criteria (Fig 3, *A*). The biggest obstacle to reaching remission in the majority of patients were the criteria “absence of symptoms” and “stable lung function” (Fig 3, *A*). When analyzing patients who were biologic-naive before dupilumab initiation, remission was achieved in 13 of 38 patients (34%) at 12 months and 6 of 24 patients (25%) at 36 months (Fig 3, *B*). In patients who were biologic-experienced before dupilumab initiation, remission was achieved in 24 of 84 patients (29%) at 12 months and in 19 of 71 patients (27%) at 36 months (Fig 3, *C*). Of the 25 patients who achieved remission at 36 months, 18 (72%) had already fulfilled the remission criteria at the 12-month time point. Patients who achieved on-treatment remission at 36 months did not differ from patients who did not achieve remission in baseline characteristics. BEC before dupilumab initiation was slightly lower in patients achieving remission; however, this was not significant when considering only patients without anti-IL-5/anti-IL-5Rα therapy during 2 months before dupilumab initiation (Table E2 in the Online Repository available at www.jaci-global.org).

### Predictors of long-term remission

Univariable logistic regression analysis did not find any significant association between baseline criteria, biomarkers, or lung function parameters before dupilumab initiation and remission at 36 months (Table E3 in the Online Repository available at www.jaci-global.org). However, several response parameters at the 3-month follow-up including increases in FEV_1_% and FVC% as well as increases in blood eosinophils were associated with remission at the 36-month time point (Table E4 in the Online Repository available at www.jaci-global.org).

## Discussion

In this study we characterized treatment patterns of biological therapies and long-term outcomes after 1 and 3 years of dupilumab therapy in a real-world cohort. We demonstrated significant improvement in exacerbations, OCS use, lung function, and symptom control in patients who continued dupilumab, with about 25% to 30% of patients achieving remission criteria at 12 and 36 months.

Compared with the dupilumab RCT QUEST and the open-label extension study TRAVERSE, our cohort was slightly older and had a higher share of patients with nasal comorbidities and former smokers.^6^^,^^8^^,^^16^ With regard to biomarkers, BEC in the subgroup of patients without recent anti-IL-5/anti-IL-5Rα treatment was similar, whereas Feno was slightly higher compared with TRAVERSE.^8^^,^^16^ Of note, patients included in the QUEST/TRAVERSE studies were biologic-naive and without maintenance OCS, whereas patients in our cohort had poor asthma control with median ACT of 14 despite high-intensity treatment at baseline, with almost 30% of patients on OCS and 70% of patients who had already undergone another biological therapy before dupilumab.^6^^,^^8^^,^^16^ Concerning high-intensity treatment, our study population was more similar to another large, real-world study of patients with severe eosinophilic asthma receiving benralizumab (XALOC-1), in which about 40% of the study cohort were biologic-experienced and about 30% received maintenance OCS therapy.^17^ However in this study, patients were older and had higher eosinophil counts, suggestive of a predominant adult-onset eosinophilic cohort compared with our mixed cohort in which both patients with early-onset and adult-onset asthma were included and the majority of patients had relevant allergies.^17^ In our study cohort, 41% of patients were former smokers with a median of 10 pack-years. A subgroup analysis of our study suggested that previous smoking did not negatively impact response to dupilumab, which is in line with the findings of BOREAS and NOTUS studies of dupilumab in patients with chronic obstructive pulmonary disease with type 2 inflammation.^18^^,^^19^

A portion of patients discontinued dupilumab during the 3-year study period, with 24% of the total cohort having discontinued treatment at 12 months and 41% having discontinued treatment at 36 months. Most patients stopped therapy during the first 12 months, the most frequent reason being insufficient treatment response. Few studies have evaluated real-world trajectories of biological treatments: In a study by Bagnasco et al,^20^ 26% of patients discontinued therapy with mepolizumab over a 3-year period. In XALOC-1, about 20% of patients stopped benralizumab therapy at the 12-month time point, most because of insufficient response.^17^ Cessation owing to insufficient treatment efficacy was also reflected in another real-life study by Fyles et al^21^ in a cohort receiving anti-IL-5/IL-5Rα therapies. Suspected side effects were another reason for discontinuation of dupilumab with the most common being respiratory and cutaneous side effects. Respiratory side effects were probably adverse events owing to exacerbations or respiratory tract infections as seen also in RCTs QUEST and VENTURE with 7% to 19% in both dupilumab and placebo groups.^6^^,^^7^ Injection site reactions were frequent in RCTs with 9% to 18% prevalence in QUEST and VENTURE and real-life studies with similar prevalences of about 14%.^6^^,^^7^^,^^22^ In 4 patients, dupilumab was discontinued owing to hypereosinophilia with organ complication—EGPA in 2 patients and eosinophilic pneumonia in 2 patients. Although transient blood hypereosinophilia was seen in several RCTs with dupilumab across different diseases in up to 14% of patients, associated side effects were rare.^23^ However, a few case reports and case series reported hypereosinophilic side effects with organ damage, including EGPA and eosinophilic pneumonia.^24^, ^25^, ^26^, ^27^ This phenomenon is currently debated as either being a side effect of dupilumab therapy or the unmasking of a previously unknown disease triggered by downtapering of OCS after the initiation of biological therapy. In our 4 cases, 3 patients were on OCS maintenance therapy, and 3 were on an anti-IL-5Rα therapy before initiation of dupilumab. In 2 patients, dupilumab was discontinued owing to arthralgia, which might have been related to dupilumab therapy.^28^, ^29^, ^30^ Although conjunctivitis was more frequently seen in patients treated with dupilumab for atopic dermatitis than in asthma studies, 2 patients discontinued treatment owing to conjunctivitis.^31^

In patients continuing dupilumab therapy for up to 36 months, there was a significant reduction in exacerbations and OCS dose as well as a significant improvement in asthma symptom control and lung function. Other real-world studies with smaller cohorts and shorter follow-up times demonstrated similar results at an earlier 12-month time point.^22^^,^^32^ Further, the data underline that the largest treatment benefits may be observed early on, often already during the first 3 months. This is in line with data from QUEST depicting large benefits already after 2 weeks of therapy.^6^ Importantly, we showed that treatment effects reached at 3 months were sustained at 12 and 36 months.

On-treatment remission has become an important therapeutic goal of targeted therapies across multiple chronic diseases, including severe asthma.^33^ Here, we defined remission as proposed by Menzies-Gow et al^13^ and the German asthma guidelines.^10^ In our total cohort, 30% and 26% of patients experienced remission at 12 and 36 months, respectively. These rates consider only patients who continued therapy and thus would be lower when considering all initiated patients. In the dupilumab open-label extension study TRAVERSE, 33% of patients experienced remission on dupilumab therapy. Compared with TRAVERSE, our study population had higher baseline treatment with a high share of previous biologics and OCS therapy.^8^ When analyzing individual remission criteria, we found that most patients in our cohort at both 12 and 36 months achieved the criteria “no exacerbations” and “no OCS,” whereas the criteria “ACT > 20 points” and “stable or better lung function” were not achieved as frequently. Importantly, remission rates were comparable between the 12- and 36-month time points, suggesting sustained treatment effects. Of note, the majority of patients in remission at 36 months already had achieved remission at the 12-month time point. To date, there are no other real-world studies examining 3-year outcomes on dupilumab. Gates et al^34^ reported that 45% of patients on dupilumab achieved remission criteria after 12 months of therapy in a cohort switched from anti-IL-5/IL-5Rα therapy. An Italian real-world study in biologic-naive patients demonstrated remission rates of 30% after 12 months and 45% after 24 months of dupilumab treatment.^9^ A Dutch real-world study examined remission on dupilumab in patients with severe asthma after 12 months and found about 30% of patients achieving remission criteria.^35^ Similar studies on remission in the GAN or the Danish severe asthma registry found remission prevalences in cohorts with severe asthma treated with biologics with slightly different criteria of 32% and 19% at 12-month time points.^36^^,^^37^ Two studies evaluating 3-year outcomes on anti-IL-5/IL-5Rα therapies found sustained clinical improvements but did not analyze asthma remission.^21^^,^^38^ A meta-analysis found a 30% remission rate using a 4-strata definition with large heterogeneity between studies.^39^

Predictors of long-term response have been identified in both clinical trials and real-world studies. Bult et al^35^ identified high BEC before dupilumab initiation and male sex as predictors of remission. Importantly, lower Feno was associated with a lower likelihood of remission in their data.^35^ In our cohort, no baseline characteristics were significantly associated with remission on dupilumab therapy. Univariate analyses suggested that patients experiencing early benefits (improvement in FEV_1_% and FVC%) within 3 months of therapy initiation were more likely to achieve remission at 36 months. In a study of the Danish asthma registry, decreasing Feno was found to be a predictor for remission in a cohort receiving anti-IL5/IL-5Rα therapy; however, decreasing Feno was not a predictor of remission in our cohort.^40^ This might be due to a rather mixed cohort of patients with both early- and adult-onset phenotypes represented in our study as well as due to previous biological therapies.

The strengths of our study are its close representation of the real-life clinical scenario, with a heavily pretreated patient cohort of an outpatient clinic specializing in management of severe asthma. It reflects real-life clinical decisions and demonstrates the trajectories of patients with severe asthma including multiple switching of biologics. Furthermore, we were able to assemble data from 3 high-volume asthma outpatient clinics. However, the study also has some limitations. Data were assessed retrospectively in a real-world setting, lacking a control group without biological treatment. In addition, clinical decisions on continuing or discontinuing treatment might have differed among physicians or participating centers; eg, there was no predefined cutoff for cessation of dupilumab therapy in the case of hypereosinophilia. Although this was a multicenter study, the number of patients in our cohort is smaller compared with the large number of participants in RCTs and open-label studies such as TRAVERSE, which may limit external validity.

In summary, dupilumab is an effective treatment option for patients with severe asthma that has sustained treatment efficacy during long-term treatment. A relevant proportion of patients achieved remission at 12 and 36 months despite previous unsuccessful treatment with biologics.

## Disclosure statement

The authors received no funding for the submitted work

Disclosure of potential conflict of interest: C. Mümmler reports personal fees and speaker fees from Sanofi and AstraZeneca, outside the submitted work. A. Lenoir reports personal fees from 10.13039/501100015086AOP Health, 10.13039/100004339Sanofi, and 10.13039/100004325AstraZeneca, outside the submitted work. J. Götschke reports personal fees and speaker fees from 10.13039/100004331Johnson & Johnson and AstraZeneca. M. Gerckens received a travel grant from Chiesi outside the submitted work. M. Kayser reports honoraria for lectures from AstraZeneca and GSK, outside the submitted work. N. Drick reports honoraria for lectures and consultation from AstraZeneca, GSK, and Sanofi, outside the submitted work. H. Suhling reports personal fees/speaker honoraria from AstraZeneca, GSK, Novartis, and Sanofi, all outside the submitted work. L. Biener reports personal fees and speaker fees from AstraZeneca, Boehringer Ingelheim, and Sanofi, all outside the submitted work. D. Skowasch reports fees for lectures or consultations from AstraZeneca, Boehringer Ingelheim, GSK, Janssen, MSD, Sanofi, all outside the submitted work. N. Kneidinger reports personal fees and speaker fees from GSK and AstraZeneca, outside the submitted work. J. Behr reports honoraria for lectures and consultation from AstraZeneca, Boehringer-Ingelheim, BMS, Gossamer Bio, Sanofi, United Therapeutics, Ferrer, and Novartis, outside the submitted work. K. Milger received speaker and/or advisor fees from AstraZeneca, Chiesi, Insmed, GSK, Novartis, Sanofi. The rest of the authors declare that they have no relevant conflicts of interest.
