# Supplementary material for: Long-term outcomes of dupilumab therapy in severe asthma: A retrospective, multicenter, real-world study
Source: J Allergy Clin Immunol Glob. 2025 Jul 8;4(4):100533. doi: 10.1016/j.jacig.2025.100533 (PMC12347924; doi:10.1016/j.jacig.2025.100533)
Supplement: Supplementary Data [file mmc1.docx]

**Supplementary Material**

**Table S1:**

| **Table S1: Characteristics of patients continuing or stopping dupilumab** | | | |
| --- | --- | --- | --- |
| *Parameter* | Continuing dupilumab | Stopping dupilumab | p-value |
| Patients, n | 95 | 65 |  |
| Age (y), median (IQR) | 57 (49-64) | 58 (47-62) | 0.61* |
| Female, n (%) | 52 (55%) | 34 (52%) | 0.87^#^ |
| BMI (kg/m^2^), median (IQR) | 27 (23-31) | 28 (24-33) | 0.29* |
| Age at diagnosis (y), median (IQR) | 30 (11-45) | 30 (10-41) | 0.58* |
| Time since asthma diagnosis (y), median (IQR) | 24 (12-37) | 22 (11-36) | 0.64* |
| Early-onset asthma (≤18Y), n (%) | 33 (35%) | 21 (32%) | 0.87^#^ |
| Adult-onset asthma (>18Y), n (%) | 62 (65%) | 44 (68%) | 0.87^#^ |
| Patients with allergies, n (%) | 66 (70%) | 46 (71%) | 0.86^#^ |
| Former smokers, n (%) | 39 (41%) | 26 (40%) | 0.99^#^ |
| *Comorbidities* |  |  |  |
| CRSwNP, n (%) | 45 (47%) | 27 (42%) | 0.52^#^ |
| Allergic rhinitis, n (%) | 31 (33%) | 24 (37%) | 0.61^#^ |
| CRSsNP, n (%) | 23 (24%) | 21 (32%) | 0.28^#^ |
| Aspirin intolerance, n (%) | 27 (28%) | 10 (15%) | 0.06^#^ |
| Atopic dermatitis, n (%) | 20 (21%) | 15 (23%) | 0.85^#^ |
| Steroid-induced side effects, n (%) | 18 (19%) | 16 (25%) | 0.43^#^ |
| COPD, n (%) | 13 (14%) | 10 (15%) | 0.82^#^ |
| *Asthma Control* |  |  |  |
| ACT, median (IQR) | 15 (10-19) | 12 (10-16) | 0.06* |
| Annual Exacerbations, median (IQR) | 1.0 (0.0-3.0) | 1.5 (0.0-4.0) | 0.25* |
| *Biomarkers* |  |  |  |
| Blood eosinophils (cells/ µl), median (IQR) | 150 (10-460) | 115 (10-340) | 0.92* |
| FeNO (ppb), median (IQR) | 41 (23-78) | 53 (23-88) | 0.70* |
| IgE (IU/ml), median (IQR) | 143 (62-334) | 150 (46-531) | 0.66* |
| *Previous therapy* |  |  |  |
| Continuous OCS therapy, n (%) | 25 (26%) | 19 (29%) | 0.72^#^ |
| OCS dose (mg), median (IQR)^~^ | 6 (5-13) | 10 (5-10) | 0.40* |
| *Previous biologic therapy* |  |  |  |
| No antibody therapy, n (%) | 24 (25%) | 21 (32%) | 0.76^+^ |
| Benralizumab, n (%) | 39 (41%) | 27 (42%) |  |
| Mepolizumab, n (%) | 19 (20%) | 10 (15%) |  |
| Omalizumab, n (%) | 11 (12%) | 5 (17%) |  |
| Reslizumab, n (%) | 2 (2%) | 2 (2%) |  |

**Table S1:** Characteristics of patients who continued dupilumab treatment for 36 months (n=95) vs. patients who discontinued dupilumab treatment (n=65). ^~^only in patients on continuous OCS therapy. Statistics: *Mann-Whitney test; ^#^ Fishers exact test; +Chi-Square test.

**Table S2:**

| **Table S2: 36 months Remission vs. Non-Remission Baseline Characteristics** | | | |
| --- | --- | --- | --- |
| *Parameter* | Remission | Non-Remission | p-value |
| Patients, n | 25 | 70 |  |
| Age (y), median (IQR) | 58 (50-64) | 57 (48-63) | 0.65* |
| Female, n (%) | 16 (64%) | 36 (51%) | 0.35^#^ |
| BMI (kg/m^2^), median (IQR) | 27 (24-30) | 28 (23-31) | 0.73* |
| Age at diagnosis (y), median (IQR) | 33 (23-47) | 26 (10-44) | 0.13* |
| Time since asthma diagnosis (y), median (IQR) | 19 (13-29) | 24 (11-40) | 0.35* |
| Early-onset asthma (<18Y), n (%) | 5 (20%) | 28 (40%) | 0.09^#^ |
| Adult-onset asthma (>18Y), n (%) | 20 (80%) | 42 (60%) | 0.09^#^ |
| Patients with allergies, n (%) | 19 (76%) | 47 (67%) | 0.46^#^ |
| Former smokers, n (%) | 8 (32%) | 31 (44%) | 0.35^#^ |
| *Comorbidities* |  |  |  |
| CRSwNP, n (%) | 15 (60%) | 30 (43%) | 0.17^#^ |
| Allergic rhinitis, n (%) | 10 (40%) | 21 (30%) | 0.46^#^ |
| CRSsNP, n (%) | 7 (28%) | 16 (23%) | 0.60^#^ |
| Aspirin intolerance, n (%) | 8 (32%) | 19 (27%) | 0.80^#^ |
| Atopic dermatitis, n (%) | 3 (12%) | 17 (24%) | 0.26^#^ |
| Steroid-induced side effects, n (%) | 6 (24%) | 12 (17%) | 0.55^#^ |
| COPD, n (%) | 3 (12%) | 10 (14%) | 0.99^#^ |
| *Asthma Control* |  |  |  |
| ACT, median (IQR) | 15 (12-20) | 15 (10-19) | 0.54* |
| Annual Exacerbations, median (IQR) | 1.0 (0.0-3.5) | 1.0 (0.0-2.0) | 0.31* |
| *Biomarkers* |  |  |  |
| Blood eosinophils (cells/ µl), median (IQR) | 60 (0-240) | 175 (10-553) | **0.05*** |
| Blood eosinophils (cells/µl) only in pts. without anti-IL-5/anti-IL5-Rα therapy 2 months prior to dupilumab initiation, median (IQR)^#^ | 220 (60-460) | 295 (158-563) | 0.26***** |
| FeNO (ppb), median (IQR) | 51 (35-87) | 39 (21-73) | 0.08* |
| IgE (IU/ml), median (IQR) | 129 (68-380) | 153 (53-334) | 0.82* |
| *Previous therapy* |  |  |  |
| Continuous OCS therapy, n (%) | 8 (32%) | 17 (24%) | 0.44^#^ |
| OCS dose (mg), median (IQR)^~^ | 8 (5-18) | 6 (4-13) | 0.68* |
| *Previous biologic therapy* |  |  |  |
| No antibody therapy, n (%) | 6 (24%) | 18 (26%) | 0.84^+^ |
| Benralizumab, n (%) | 12 (48%) | 27 (39%) |  |
| Mepolizumab, n (%) | 4 (16%) | 15 (21%) |  |
| Omalizumab, n (%) | 3 (12%) | 8 (11%) |  |
| Reslizumab, n (%) | 0 | 2 (3%) |  |

**Table S2:** Characteristics of patients who achieved remission or not at 36 months after dupilumab initiation. ^~^only in patients on continuous OCS therapy. Statistics: *Mann-Whitney test; ^#^ Fishers exact test; +Chi-Square test.

**Table S3:**

| Logistic regression on 36 months remission outcome | | |
| --- | --- | --- |
| Univariable | **OR (95 % CI)** | **P** |
| Age of Onset < 18 Years | 0.38 (0.13-1.12) | 0.08 |
| FeNO > 25 ppB | 1.65 (0.61-4.47) | 0.32 |
| feNO > 50 PPB | 1.20 (0.47-3.08) | 0.70 |
| BEC > 150 /µl | 1.00 (0.22-4.63) | 0.99 |
| BEC > 300 /µl | 1.24 (0.32-4.72) | 0.10 |
| IGE > 100 IU/ml | 0.94 (0.35-2.52) | 0.91 |

**Table S3:** Univariable logistic regression of baseline characteristics as predictors for achieving a 36 months remission outcome. Abbreviations: CI confidence interval.

**Table S4:**

| Logistic regression on 36 months remission outcome | | |
| --- | --- | --- |
| Univariable | **OR (95 % CI)** | **P** |
| Δ ACT per point | 1.11 (1.00-1.23) | 0.06 |
| Δ BEC per 100 Eos/µl | **1.10 (1.01-1.20)** | **0.03** |
| Δ Feno per 10 ppb | 0.99 (0.88-1.11) | 0.84 |
| Δ FEV1 per 100 ml | **1.34 (1.15-1.56)** | **<0.01** |
| Δ FVC per 100 ml | **1.29 (1.13-1.48)** | **<0.01** |
| Δ RV per 100 ml | 0.97 (0.89-1.05) | 0.40 |

**Table S4:** Univariable logistic regression of differences at 3 months compared to baseline as predictors for achieving a 36 months remission outcome. Abbreviations: CI confidence interval.

**Figure S1:**

**Figure S1:** Subgroup analysis of clinical outcome parameters of patients who continued dupilumab therapy 36 months after initiation. A) subgroup of ex-smokers (39 patients). B) subgroup of patients with CRSwNP (45 patients). C) subgroup of patients with early-onset asthma (33 patients). D) subgroup of patients with late-onset asthma (62 patients). Abbreviations: ACT: asthma control test. OCS: oral corticosteroid. FEV1%: percent predicted of forced expiratory volume in 1 second. Statistics: A mixed-effects model was used for statistical analysis. ** P < .01; *** P < .001; **** P < .0001; ns not significant.
